# Metformin attenuates TBHP-induced oxidative injury in human lens epithelial cells and is associated with SIRT1/FOXO1-related autophagy

**DOI:** 10.1371/journal.pone.0346822

**Published:** 2026-04-07

**Authors:** Zhuxuan Yan, Wei Chi, Zhenguo Yan, Hanrui Wang

**Affiliations:** 1 The First Clinical Medical College of Lanzhou University, Lanzhou, Gansu, China; 2 Shenzhen Eye Hospital, Shenzhen Eye Medical Center, Southern Medical University, Shenzhen, China; 3 Lanzhou Huaxia Eye Hospital, Lanzhou, Gansu, China; Dana Farber Cancer Institute, Harvard Medical School, UNITED STATES OF AMERICA

## Abstract

Metformin (MET), a first-line antidiabetic drug, has been increasingly implicated in cellular protection under oxidative stress, yet its mechanisms in lens epithelial cells (LECs) remain incompletely defined. Using a tert-butyl hydroperoxide (TBHP)-induced acute oxidative injury model in human HLE-B3 cells, we investigated whether SIRT1/FOXO1-related autophagy contributes to MET-associated cytoprotection. MET pretreatment reduced intracellular reactive oxygen species, preserved antioxidant defenses, improved cell viability, and decreased apoptosis after TBHP challenge. MET also enhanced autophagy markers and, under lysosomal blockade with chloroquine or bafilomycin A1, showed LC3-II/p62 changes consistent with increased autophagic flux. Pharmacologic inhibition of SIRT1 (EX-527) or early-stage autophagy (3-methyladenine) partially attenuated MET-associated improvements across oxidative stress and survival endpoints, supporting a role for SIRT1/FOXO1-related autophagy in this response. Although limited to an in vitro setting and pharmacological perturbation, these findings suggest that MET may mitigate oxidative injury in lens epithelium, highlighting SIRT1/FOXO1-autophagy as a potential pathway relevant to oxidative stress processes in cataractogenesis.

## Introduction

Age-related cataract (ARC) is the leading cause of blindness globally [1]. Its pathogenesis is multifactorial, but oxidative injury to lens epithelial cells (LECs) is a central driver [2]. Reactive oxygen species (ROS) can overwhelm endogenous defenses, precipitating protein oxidation and crosslinking, DNA damage, and apoptosis, which in turn promote lens opacification and vision loss [3]. Although cataract surgery effectively restores vision, its reach is limited by surgical risk, contraindications, and socioeconomic barriers [4]. Mechanistic insights that enable pharmacologic delay or prevention of cataractogenesis are therefore urgently needed.

Metformin (MET), a first-line antidiabetic drug, has attracted interest for antioxidant and cytoprotective actions beyond glycemic control [5,6]. In ocular contexts, MET mitigates oxidative injury and enhances lysosomal/autophagic function, with studies suggesting a potential to slow cataract progression [7–9]. Prior work has largely emphasized AMP-activated protein kinase (AMPK)–dependent autophagy, but whether MET also engages the silent information regulator 1 (SIRT1)/forkhead box O1 (FOXO1) axis in LECs remains unclear.

Autophagy is a conserved quality-control pathway that removes damaged organelles and misfolded proteins, thereby sustaining redox homeostasis under stress [10]. The NAD^+^-dependent deacetylase SIRT1 modulates autophagy in part by deacetylating transcription factors such as FOXO1 and promoting stress-adaptive transcriptional programs [11]. In the lens, such regulation could couple oxidative-stress sensing to restoration of proteostasis and cell survival.

Here, we asked whether MET pretreatment elicits an EX-527–sensitive SIRT1/FOXO1 stress response that is accompanied by autophagy marker changes/flux-consistent signatures and improved survival under an acute TBHP oxidative injury paradigm in human HLE-B3 cells. Using TBHP as a model of global oxidative injury, we examined MET-associated changes in SIRT1/FOXO1 signaling, canonical autophagy markers (Beclin-1, LC3B, p62), and lysosomal-blockade readouts together with oxidative stress and apoptosis endpoints. Our objective was to delineate cytoprotective mechanisms in lens epithelium under an acute injury framework; endpoints related to cellular senescence or mitochondria-specific ROS paradigms were considered beyond the prespecified scope of this study.

## Materials and methods

### Cell culture

The human lens epithelial cell line HLE-B3 (SV40-transformed; Procell, China; Cat# CL-0913) was maintained in Minimum Essential Medium with non-essential amino acids (MEM/NEAA; Gibco, USA; Cat# 10370088) supplemented with 10% fetal bovine serum (FBS; Gibco, USA; Cat# A5256701) and 1% penicillin–streptomycin (P/S; Gibco, USA; Cat# 15140148). Cells were cultured at 37 °C in a humidified incubator with 5% CO_2_, and complete medium was refreshed every other day. Monolayers at ~70–80% confluence were washed with phosphate-buffered saline (PBS; Sigma-Aldrich, USA; Cat# P2272) and passaged using 0.25% trypsin (Sigma-Aldrich, USA; Cat# T4049). Unless otherwise specified, all experiments were initiated using logarithmically growing cells at ~70–80% confluence.

### Ethics statement

This study did not involve human participants, human data, or animal experiments. The experiments were conducted using the commercially available human lens epithelial cell line (HLE-B3). Therefore, ethical approval and informed consent were not required.

### Oxidative stress injury model and experimental groups

Oxidative stress was induced using tert-butyl hydroperoxide (TBHP; Macklin, China; Cat# B802372). Pilot Cell Counting Kit-8 (CCK-8) titrations (100–800 μM, 4 h) identified 500 μM TBHP for 4 h as a reproducible sublethal condition (~50% viability) for subsequent mechanistic experiments. Unless otherwise stated, the experimental timeline was as follows: cells were seeded and allowed to adhere overnight; metformin (MET; Sigma-Aldrich, USA; Cat# D150959) pretreatment was applied for 24 h; where indicated, inhibitors were added before the oxidative challenge; cells were then exposed to TBHP for 4 h and processed immediately for endpoint assays or harvest.

All TBHP exposures were performed in complete medium containing 10% FBS (no serum starvation). After the 24-h MET pretreatment, the medium was aspirated and replaced with fresh complete medium containing TBHP (500 μM) with or without inhibitors (MET was not present during the 4-h TBHP exposure). For pathway interrogation, cells were pretreated with MET (0.5 mM, 24 h) and then treated with 3-methyladenine (3-MA; TargetMol, China; Cat# T1879; 5 mM, 1 h) or EX-527 (TargetMol, China; Cat# T6111; 10 μM, 30 min) prior to TBHP exposure. For autophagic-flux experiments, chloroquine (CQ; TargetMol, China; Cat# T8689; 50 μM) or bafilomycin A1 (BafA1; TargetMol, China; Cat# T6740; 100 nM) was applied during the TBHP challenge (CQ for the final 3 h; BafA1 for the final 1 h) prior to harvest to block lysosomal degradation and facilitate flux interpretation. Inhibitors were prepared in dimethyl sulfoxide (DMSO; Sigma-Aldrich, USA; Cat# D2650), and vehicle-matched controls were included with an identical final DMSO concentration across groups.

### Cell viability assay

Cell viability was assessed using CCK-8 (MedChemExpress, USA; Cat# HY-K0301) according to the manufacturer’s instructions. HLE-B3 cells were seeded at 1 × 10^4^ cells/well into 96-well plates (100 μL complete medium) and allowed to adhere overnight. To establish oxidative injury conditions, cells were exposed to TBHP (100–800 μM) for 4 h. To evaluate potential cytotoxicity of MET, cells were treated with MET (0.5–4.0 mM) for 24 h. For protection experiments, cells were pretreated with MET for 24 h; the pretreatment medium was removed and replaced with fresh complete medium containing TBHP (500 μM) for 4 h. After treatments, medium was replaced with fresh complete medium containing 10% (v/v) CCK-8 working solution and incubated at 37 °C for 2 h. Absorbance at 450 nm was measured using a microplate reader; background (medium + CCK-8 without cells) was subtracted, and viability was expressed as a percentage of the untreated control. Each condition included five technical replicate wells per independent experiment, and experiment-level means were used for statistical analysis.

### Intracellular ROS measurement

Intracellular reactive oxygen species (ROS) were assessed using a fluorometric ROS assay kit (Elabscience, China; Cat# E-BC-F005) according to the manufacturer’s instructions. HLE-B3 cells were seeded at 2 × 10^4^ cells/well into 24-well plates and treated as indicated. After treatment, adherent cells were gently washed 2–3 times with Reagent 1 working solution (kit buffer). Cells were then incubated with the ROS probe working solution (final 5 μM, prepared by diluting Reagent 2 in Reagent 1 working solution) at 37 °C in the dark for 30 min. Excess probe was removed by washing 2–3 times with Reagent 1 working solution, and images were acquired immediately using a fluorescence microscope with a Cy3/Texas Red/RFP filter set (Ex ~ 518 nm, Em ~ 610 nm) under identical exposure settings across groups. Fluorescence was quantified in ImageJ after background subtraction from randomly selected fields; technical fields were averaged within each biological replicate, and replicate-level means were used for statistical analysis.

### Malondialdehyde (MDA) assay

Cellular MDA was quantified using an enhanced TBA-based MDA colorimetric assay kit (Elabscience, China; Cat# E-BC-K814-M) according to the manufacturer’s instructions. After treatments, at least 3 × 10^6^ cells were collected and lysed in 0.5 mL lysis solution with thorough mixing for 3 min. An aliquot was centrifuged at 4 °C (10,000 × g, 10 min), and the supernatant was used for protein determination using a BCA assay kit (Beyotime, China; Cat# P0010). Remaining lysates were reacted with TBA working reagents, heated at 95–100 °C for 40 min, cooled, centrifuged, and absorbance was measured at 532 nm. Reagent blanks were subtracted; turbid samples were re-centrifuged until clear prior to reading. MDA concentrations were calculated from a standard curve and expressed as nmol/mg protein.

### Superoxide dismutase (SOD) activity assay

Total SOD activity was measured using a WST-1–based colorimetric assay kit (Elabscience, China; Cat# E-BC-K020-M). After treatments, 1 × 10^6^ cells were collected, homogenized in 300–500 μL PBS, and centrifuged at 4 °C (10,000 × g, 10 min). Supernatants were kept on ice for analysis and normalized by protein concentration (BCA; Beyotime, China; Cat# P0010). Sample dilutions were pre-optimized to ensure inhibition within the recommended range (25%–65%). Assays were assembled per the kit protocol, incubated at 37 °C for 20 min, and absorbance was read at 450 nm. SOD activity was calculated using the manufacturer’s equation and expressed as U/mg protein.

### Myeloperoxidase (MPO) activity assay

MPO activity was measured using a colorimetric kit (Elabscience, China; Cat# E-BC-K074-S) following the manufacturer’s instructions. After treatment, 2 × 10^6^ cells were homogenized in the kit working solution, clarified by centrifugation to obtain a clear supernatant, and assayed according to the protocol. Absorbance was read at 460 nm, and MPO activity was calculated using the kit-provided equation and expressed as U/10^6^ cells.

### Glutathione (GSH) assay

GSH was quantified colorimetrically using a commercial kit (Invitrogen, USA; Cat# EIAGSHC) following the manufacturer’s instructions. After treatment, 1 × 10^6^ cells were rinsed with PBS and deproteinized in 5% (w/v) sulfosalicylic acid (SSA) (5-sulfosalicylic acid; Beyotime, China; Cat# Y230797). Deproteinized lysates were incubated for 10 min at 4 °C and centrifuged at 14,000 × g for 10 min (4 °C). Supernatants were diluted with kit Assay Buffer to a final 1% SSA, further diluted with Sample Diluent as needed, and loaded (50 μL/well). Colorimetric Detection Reagent and Reaction Mixture were added (25 μL each), plates were incubated for 20 min at room temperature, and absorbance was read at 405 nm. GSH concentrations were calculated from a standard curve and normalized to protein content (nmol/mg protein).

### Apoptosis detection

Apoptosis was assessed using a one-step TUNEL assay kit (Elabscience, China; Cat# E-CK-A320). HLE-B3 cells were seeded at 2 × 10^4^ cells/well into 24-well plates (on chamber slides/coverslips) and treated as indicated. Cells were fixed with 4% paraformaldehyde (Servicebio, China; Cat# G1101) for 15 min at room temperature, rinsed with PBS, and permeabilized with 0.2% Triton X-100 (Servicebio, China; Cat# GC204003) for 5 min. Slides were incubated with the TUNEL reaction mixture at 37 °C in the dark for 90 min, washed with PBS, and counterstained with DAPI (MedChemExpress, USA; Cat# HY-D2868). Images were acquired using identical microscope settings across groups. Random fields were analyzed in ImageJ, and apoptosis was expressed as the percentage of TUNEL-positive nuclei among total DAPI-positive nuclei.

### Autophagy level assessment

Autophagic vacuoles were assessed using a monodansylcadaverine (MDC) staining kit (Beyotime, China; Cat# C3018S) according to the manufacturer’s protocol. HLE-B3 cells were seeded at 2 × 10^4^ cells/well into 6-well plates and treated as indicated. Cells were incubated with MDC working solution at 37 °C for 30 min in the dark, rinsed with PBS, and imaged by epifluorescence microscopy (Ex ~ 335 nm/Em ~ 512 nm) using identical acquisition settings across groups. MDC signal was quantified in ImageJ from randomly selected fields as puncta number per cell and/or mean fluorescence intensity after background subtraction using a predefined thresholding pipeline.

### Immunofluorescence staining

HLE-B3 cells were seeded at 2 × 10^4^ cells/well into 24-well plates on coverslips and treated as indicated. Cells were fixed with 4% paraformaldehyde (Servicebio, China; Cat# G1101) for 15 min and permeabilized with 0.2% Triton X-100 (Servicebio, China; Cat# GC204003) for 10 min. After blocking with 2.5% normal goat serum (Invitrogen, USA; Cat# R37624) for 1 h at room temperature, cells were incubated overnight at 4 °C with primary antibodies against Beclin 1 (Affinity Biosciences, China; Cat# AF5128), LC3B (Affinity Biosciences, China; Cat# AF4650), p62/SQSTM1 (Abcam, UK; Cat# ab109012), SIRT1 (Abcam, UK; Cat# ab189494), and FOXO1 (Abcam, UK; Cat# ab179450) (all 1:200). After PBS washes, cells were incubated for 1 h at room temperature in the dark with goat anti-rabbit IgG (H + L) secondary antibodies conjugated to Alexa Fluor 488 (Invitrogen, USA; Cat# A-11008) or Alexa Fluor 594 (Invitrogen, USA; Cat# A-11012) (both 1 μg/mL). Nuclei were counterstained with DAPI. Images were acquired under identical microscope settings for all groups. Fluorescence was quantified in ImageJ (National Institutes of Health, USA) on randomly selected fields using a prespecified, uniform background-subtraction workflow and reported as mean fluorescence intensity per cell.

### Western blotting

HLE-B3 cells were seeded at 5 × 10^5^ cells/well into 6-well plates and treated as indicated. Cells were lysed in RIPA buffer (Beyotime, China; Cat# P0013E) supplemented with PMSF (Servicebio, China; Cat# G2008). Clarified lysates were obtained by centrifugation (12,000 × g, 10 min, 4 °C), and protein concentrations were determined using a BCA assay (Beyotime, China; Cat# P0010). Equal amounts of protein (40 μg) were separated by SDS-PAGE and transferred onto PVDF membranes (Millipore, USA; Cat# MCHL10S03, 0.45 μm). Membranes were blocked with 5% non-fat milk in TBST (Servicebio, China; Cat# G0004) for 1 h at room temperature and incubated overnight at 4 °C with rabbit primary antibodies against Beclin 1 (1:1000), LC3B (1:1000), p62/SQSTM1 (1:10,000), SIRT1 (1:1000), FOXO1 (1:1000), acetyl-FOXO1A (Lys294) (Affinity Biosciences, China; Cat# AF2305; 1:1000), and GAPDH (Affinity Biosciences, China; Cat# T0004). After washing, membranes were incubated with HRP-conjugated goat anti-rabbit IgG (H + L) (Affinity Biosciences, China; Cat# S0001; 1:3000) for 1 h at room temperature. Bands were visualized using ECL substrate (Servicebio, China; Cat# G2161) and imaged on a chemiluminescence detection system. Densitometry was performed in ImageJ (National Institutes of Health, USA). For 4-group analyses, the LC3B-II/I ratio was used to assess LC3 conversion; for 5-group autophagic-flux assays, LC3B-II/GAPDH and p62/GAPDH were quantified. Other targets were normalized to GAPDH and expressed relative to control.

### Statistical analysis

For CCK-8 assays, each independent experiment included five technical replicate wells per condition; technical replicates were averaged within each experiment, and the experiment-level mean was used for statistical inference. Data are presented as mean ± SD from independent biological replicates (n is indicated in the figure legends). Normality and homogeneity of variance were assessed using the Shapiro–Wilk and Brown–Forsythe tests, respectively. For comparisons involving ≥3 groups, one-way ANOVA with Tukey’s post hoc test was used when assumptions were met; otherwise, Welch’s one-way ANOVA was applied. Two-group comparisons were performed using two-tailed unpaired t-tests with Welch’s correction when variances were unequal. Exact P values are reported where space permits, and statistical significance was set at P < 0.05. Analyses were performed using GraphPad Prism.

## Results

### Establishment of oxidative stress model and effects of MET on LEC viability

**Fig 1:**
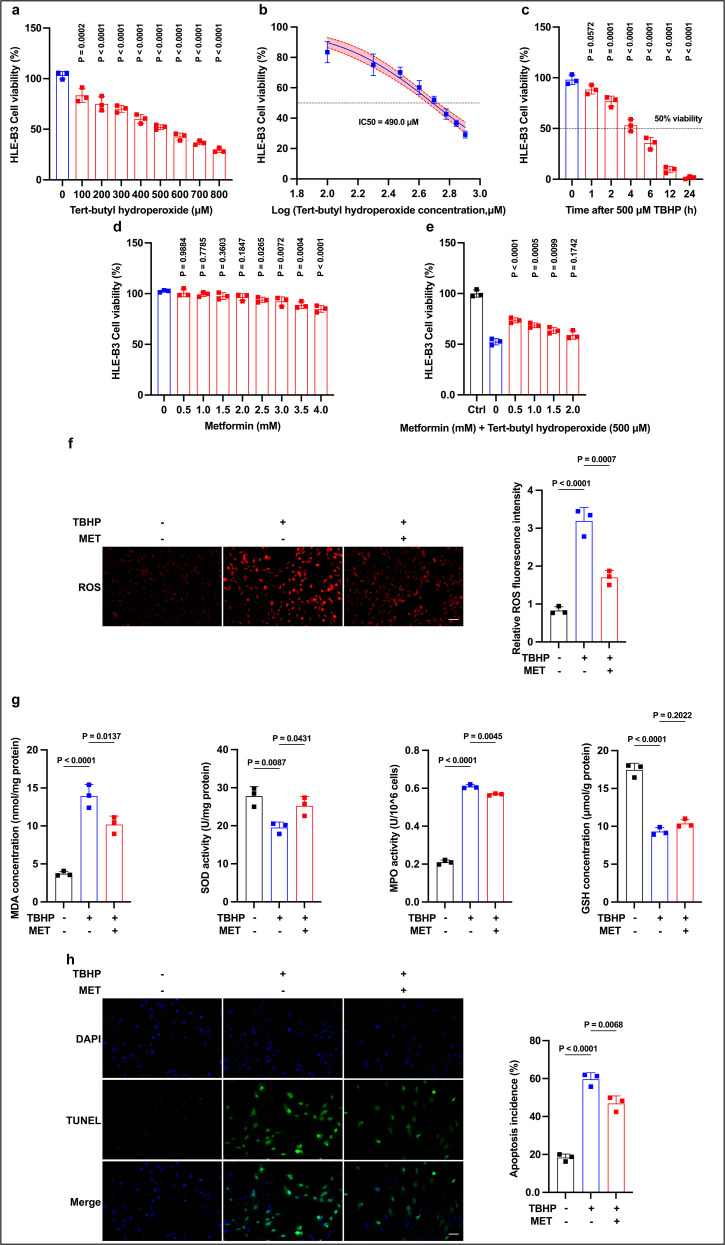
TBHP model in HLE-B3 and cytoprotection by MET. (a,b) Dose-response (4 h) to TBHP with nonlinear fit (IC50 ≈ 490 μM); 500 μM was selected. (c) Time-course (1–24 h) at 500 μM identifies 4 h (~50% survival) as a robust sublethal exposure. (d) MET alone (0–4 mM, 24 h) is non-toxic ≤ 2.0 mM; ≥ 2.5 mM reduces viability. (e) MET pretreatment (0–2.0 mM, 24 h) before TBHP (500 μM, 4 h) improves survival vs TBHP alone; 0.5 mM is most protective. (f) ROS: TBHP increases intracellular ROS; MET reduces it (rep. images and quant.; Scale bar, 50 μm). (g) Biochemistry: TBHP elevates MDA/MPO and lowers SOD/GSH; MET reverses MDA/MPO and restores SOD, while GSH remains largely unchanged. (h) TUNEL (DAPI nuclei): TBHP increases apoptosis; MET reduces it (rep. images and quant.; Scale bar, 50 μm). Data are mean ± SD (n = 3). Stats: one-way ANOVA (Tukey). Exact P values are shown in panels.

To establish an in vitro oxidative-stress model, HLE-B3 cells were treated with increasing concentrations of TBHP for 4 h, yielding a clear dose–response curve with an IC50 of ~490 μM (Fig 1a,b). At 500 μM, a 1–24 h time-course demonstrated that 4 h consistently produced ~50% survival, whereas longer exposures caused excessive lethality, supporting 4 h as a robust sublethal condition for mechanistic assays (Fig 1c). Next, MET alone was tested: 0.5–2.0 mM MET showed no detectable cytotoxicity, whereas ≥2.5 mM significantly reduced viability (Fig 1d). Pretreatment with MET (0.5–2.0 mM, 24 h) before TBHP significantly improved survival versus TBHP alone, with 0.5 mM providing the greatest protection (Fig 1e). Collectively, these data indicate that 500 μM TBHP for 4 h is the optimal oxidative-stress condition in HLE-B3 cells and that MET enhances cell survival under oxidative stress, with 0.5 mM providing maximal benefit.

### Effects of MET on oxidative stress markers in LECs

We next examined oxidative stress markers. Using a fluorometric ROS probe assay, TBHP markedly increased intracellular ROS relative to control, whereas MET pretreatment significantly suppressed this increase (Fig 1f). Consistent with these findings, TBHP increased oxidative injury indices (lipid peroxidation and peroxidase activity; MDA and MPO activity) and reduced antioxidant defenses (SOD activity and GSH levels). MET pretreatment largely reversed these changes: MDA and MPO activity decreased and SOD activity increased relative to TBHP alone; however, GSH levels were not significantly restored under our experimental conditions (Fig 1g). Together, these results indicate that MET attenuates TBHP-induced oxidative stress in HLE-B3 cells, with concordant effects across ROS imaging and biochemical assays.

### Effects of MET on apoptosis of LECs under oxidative stress

We next examined whether MET mitigates apoptosis triggered by oxidative stress. TUNEL analysis showed that TBHP exposure increased the percentage of TUNEL-positive nuclei relative to control, whereas MET pretreatment significantly reduced apoptosis compared with TBHP alone (Fig 1h). Quantitatively, MET restored TUNEL positivity toward the baseline level across independent experiments (statistics reported in the figure legend). These findings indicate that suppression of apoptosis contributes to MET’s overall cytoprotective effect in oxidatively stressed LECs.

### Effects of MET on autophagy markers in oxidatively stressed LECs

**Fig 2:**
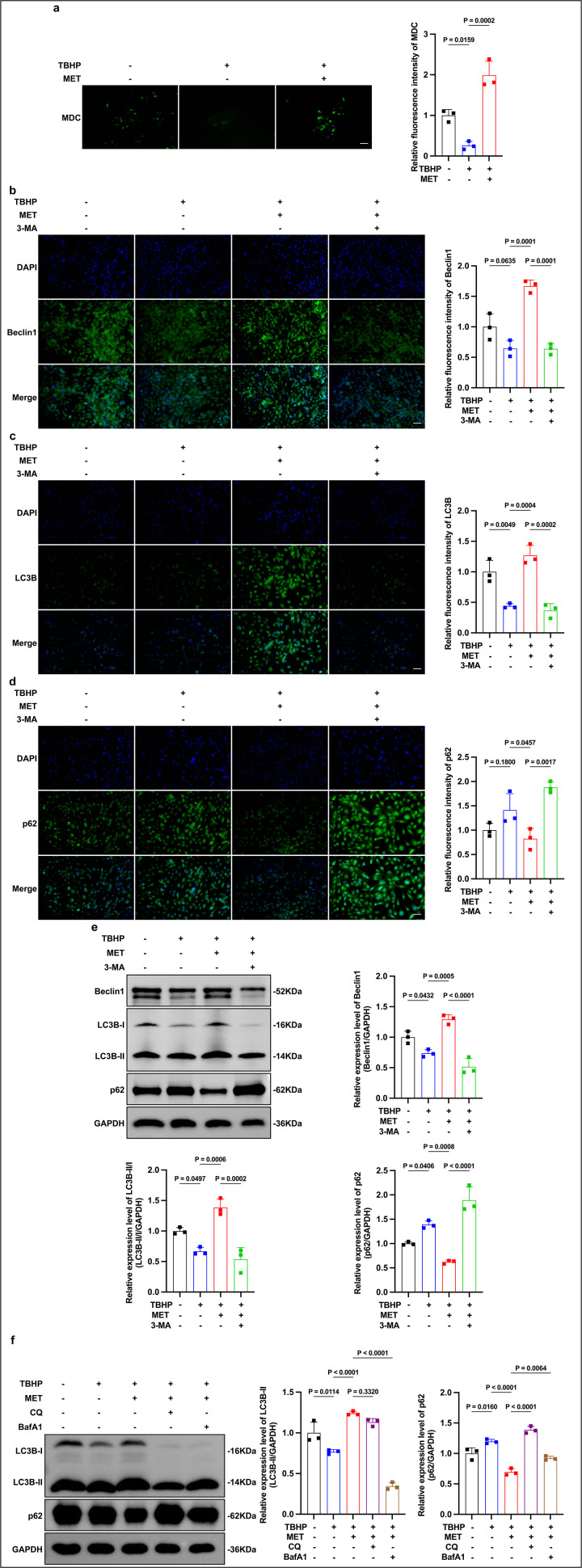
MET promotes autophagy and enhances autophagic flux under TBHP. (a) MDC puncta: TBHP reduces puncta; MET restores/increases puncta (rep. images and quant.; Scale bar, 50 μm). (b-d) IF for Beclin-1, LC3B, p62: TBHP lowers Beclin-1/LC3B and elevates p62; MET reverses these trends; 3-MA attenuates MET-induced changes (rep. images and quant.; Scale bar, 50 μm). (e) WB with densitometry (to GAPDH): TBHP decreases Beclin-1 and LC3B-II/I and increases p62; MET increases Beclin-1 and LC3B-II/I and decreases p62; 3-MA blunts these effects. (f) Flux assay with lysosomal blockade: vs TBHP, MET increases LC3B-II and reduces p62; with CQ, LC3B-II remains high and p62 accumulates; with BafA1, p62 accumulates while LC3B-II shows no further rise under short exposure—consistent with degradation blockade during active autophagosome formation. Data are mean ± SD (n = 3). Stats: one-way ANOVA (Tukey). Exact P values are shown in panels.

To probe whether MET modulates autophagy under oxidative stress, we first assessed MDC labeling of acidic/autophagic vacuoles. TBHP reduced MDC puncta, whereas MET pretreatment restored or increased puncta relative to TBHP alone (Fig 2a). Because MDC labeling lacks specificity, we next examined canonical autophagy markers by immunofluorescence (IF) and immunoblotting (WB), and tested pharmacologic sensitivity with 3-MA. IF showed that TBHP decreased Beclin-1 and LC3B signals while increasing p62, consistent with suppressed autophagy; MET pretreatment reversed these trends, and 3-MA attenuated the MET-induced changes (Fig 2b-d). WB analyses corroborated the imaging data: TBHP lowered Beclin-1 and the LC3B-II/I ratio and elevated p62, whereas MET increased Beclin-1, raised LC3B-II/I, and reduced p62 compared with TBHP; co-treatment with 3-MA blunted these effects (Fig 2e). Collectively, these marker-based and inhibitor-sensitive changes are consistent with MET being associated with enhanced autophagy signaling under oxidative stress, aligning with its cytoprotective profile. Because pathway interrogation relied on pharmacologic inhibition, these data should be interpreted as supportive rather than definitive for causal assignment.

### MET enhances autophagic flux under oxidative stress

To probe autophagic process dynamics beyond static markers, we applied short-term lysosomal blockade during the TBHP challenge. Relative to TBHP alone, MET pretreatment increased LC3B-II and reduced p62 (Fig 2f), consistent with enhanced autophagy marker conversion under oxidative stress. Upon chloroquine (CQ) treatment, p62 accumulated while LC3B-II remained elevated, indicating impaired cargo clearance during active autophagosome turnover. With brief bafilomycin A1 (BafA1) exposure, p62 also increased, whereas LC3B-II did not further rise; this pattern can occur depending on inhibitor timing/kinetics and LC3 turnover and therefore should not be over-interpreted as a single-marker determinant. Accordingly, we interpret the MET-associated LC3B conversion together with p62 accumulation upon lysosomal blockade as a flux-consistent signature, while acknowledging that reporter-based assays (e.g., tandem LC3) would provide stronger process-level validation.

### Involvement of autophagy in the antioxidative effects of MET

**Fig 3:**
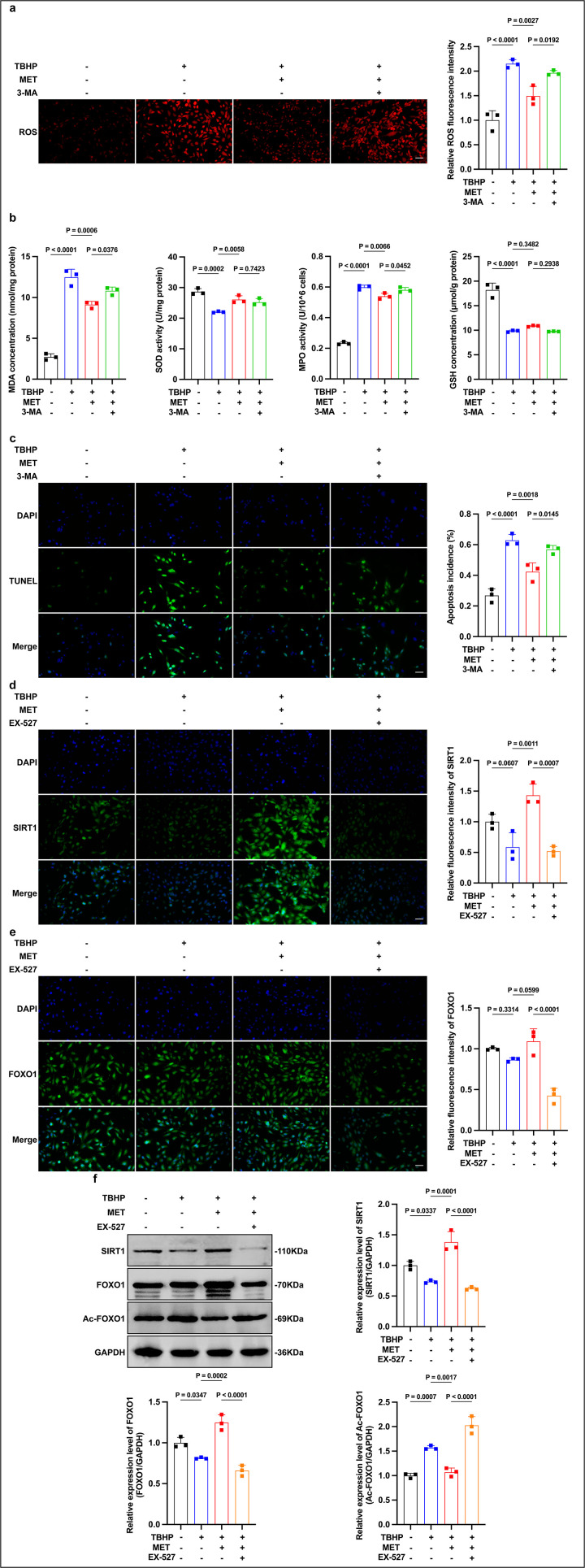
Autophagy contributes to MET’s antioxidative/anti-apoptotic actions; MET activates SIRT1/FOXO1. (a) ROS: TBHP increases ROS; MET pretreatment reduces it; 3-MA partially reverses the MET effect (rep. images and quant.; Scale bar, 50 μm). (b) Lipid peroxidation/peroxidase: TBHP elevates MDA/MPO; MET lowers both; 3-MA raises these indices vs MET. (c) TUNEL (DAPI): TBHP increases apoptosis; MET reduces it; 3-MA partially negates protection (rep. images and quant.; Scale bar, 50 μm). (d) SIRT1 IF (rep. images and MFI): TBHP reduces signal; MET restores/increases it (Scale bar, 50 μm). (e) FOXO1 IF shows a similar pattern (Scale bar, 50 μm). (f) WB with densitometry (to GAPDH): TBHP decreases SIRT1 and FOXO1 and increases Ac-FOXO1; MET increases SIRT1/FOXO1 and reduces Ac-FOXO1. Data are mean ± SD (n = 3). Stats: one-way ANOVA (Tukey). Exact P values are shown in panels.

To determine whether autophagy contributes to MET’s antioxidative effects, we examined ROS and biochemical readouts in the presence of the early-stage autophagy inhibitor 3-MA. TBHP increased intracellular ROS relative to control, whereas MET pretreatment significantly suppressed this increase; co-treatment with 3-MA partially reversed the ROS reduction produced by MET (Fig 3a). A similar pattern was observed for lipid peroxidation: TBHP elevated MDA and peroxidase activity (MPO-kit readout), MET lowered both, and 3-MA raised them again relative to MET alone (Fig 3b). In contrast, GSH content remained low and was not significantly restored by MET. Together, these observations suggest a differential antioxidant profile in which MET improves enzymatic defenses (e.g., SOD) and lowers oxidative burden (ROS, MDA, and peroxidase activity), but exerts limited effects on glutathione homeostasis in this setting.

### Involvement of autophagy in the anti-apoptotic effects of MET

We next asked whether autophagy contributes to the anti-apoptotic action of MET. TUNEL quantification showed that TBHP increased the percentage of apoptotic nuclei relative to control, whereas MET pretreatment significantly reduced apoptosis (Fig 3c). Importantly, co-treatment with the early autophagy inhibitor 3-MA partially reversed the MET-induced reduction in apoptosis. Across independent experiments, the 3-MA group exhibited a higher TUNEL index than MET alone (statistics provided in the figure legend). These data indicate that MET’s anti-apoptotic effect under oxidative stress is at least partly autophagy dependent.

### Effects of MET on SIRT1/FOXO1 signaling under oxidative stress

To clarify how MET links to autophagy and cytoprotection, we interrogated SIRT1/FOXO1 signaling in TBHP-injured HLE-B3 cells with MET pretreatment and, where indicated, the SIRT1 inhibitor EX-527. Immunofluorescence showed that TBHP reduced SIRT1 and FOXO1 fluorescence intensities relative to control, whereas MET pretreatment restored both; co-treatment with EX-527 attenuated these increases (Fig 3d,e). Quantification of mean fluorescence intensity showed the same pattern across replicates, indicating a consistent, inhibitor-sensitive upregulation of SIRT1 and FOXO1 by MET. Immunoblotting corroborated the imaging results: TBHP decreased SIRT1 and FOXO1 and increased acetylated FOXO1 (Ac-FOXO1), whereas MET increased SIRT1, restored FOXO1, and lowered Ac-FOXO1 compared with TBHP alone; these MET-induced changes were largely abolished by EX-527 (Fig 3f). Densitometry (normalized to GAPDH) confirmed higher SIRT1/GAPDH and FOXO1/GAPDH levels together with a reduced Ac-FOXO1/FOXO1 ratio in the MET group versus TBHP, with reversal upon EX-527. Together, these data indicate that MET activates SIRT1 and promotes FOXO1 deacetylation under oxidative stress, consistent with engagement of SIRT1/FOXO1-linked stress-response and autophagy programs.

### Involvement of SIRT1/FOXO1 signaling in the protective effects of MET

**Fig 4:**
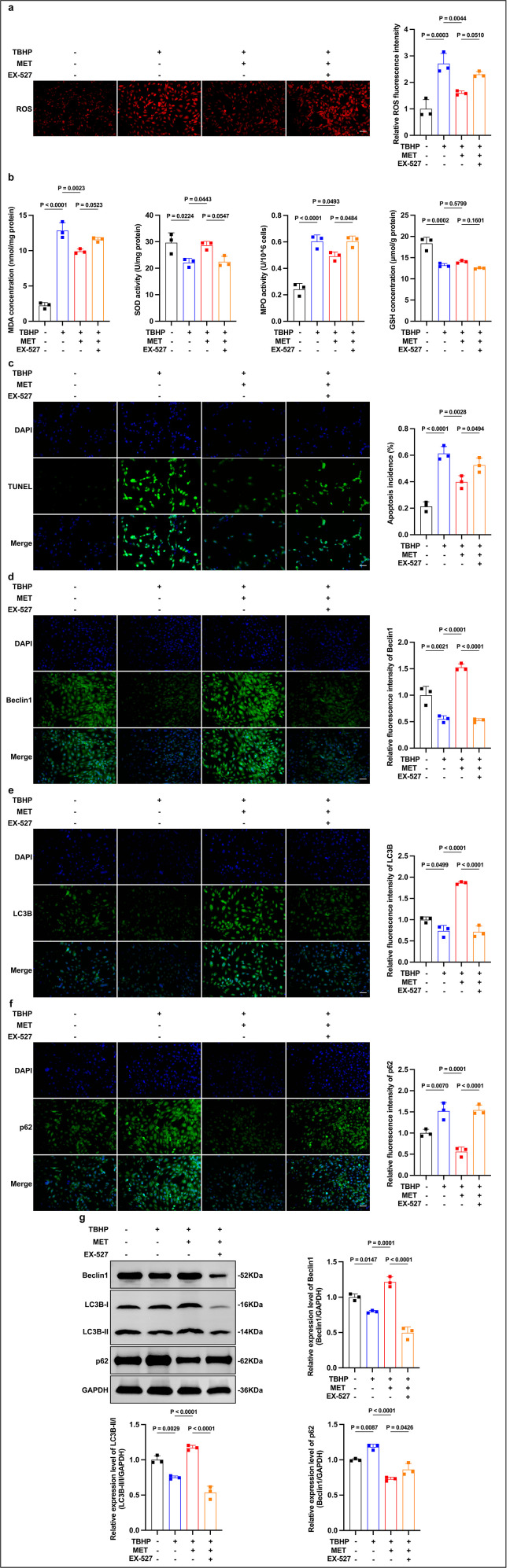
Pharmacological inhibition of SIRT1 attenuates MET-associated protection and pro-autophagy under TBHP. (a) ROS: EX-527 partially abrogates the MET-induced reduction (rep. images and quant.; Scale bar, 50 μm). (b) Biochemistry: under TBHP, MET lowers MDA/MPO; EX-527 increases both vs MET (SOD/GSH show no significant difference between MET and MET + EX-527). (c) TUNEL (DAPI): EX-527 diminishes the anti-apoptotic effect of MET (rep. images and quant.; Scale bar, 50 μm). (d-f) IF of Beclin-1, LC3B, p62: the MET-associated pattern (higher Beclin-1 and LC3B, lower p62) is attenuated by EX-527 (Scale bar, 50 μm). (g) WB with densitometry: TBHP decreases Beclin-1 and LC3B-II/I and increases p62; MET reverses these changes; EX-527 blunts the MET effects. Data are mean ± SD (n = 3). Stats: one-way ANOVA (Tukey). Exact P values are shown in panels.

We next tested whether SIRT1/FOXO1 mediates MET’s protection against oxidative stress and apoptosis in LECs. TBHP elevated intracellular ROS relative to control, whereas MET pretreatment reduced ROS; co-treatment with EX-527 partially abrogated this reduction, resulting in higher ROS in the MET + EX-527 group than with MET alone (Fig 4a). Consistently, MET lowered MDA and peroxidase activity (MPO-kit readout) under TBHP, and EX-527 increased both relative to MET alone (although remaining below the TBHP-alone levels); by contrast, SOD activity and GSH levels did not differ significantly between MET and MET + EX-527 (Fig 4b). In line with these effects, TUNEL analysis showed that EX-527 also diminished MET’s anti-apoptotic effect such that apoptosis in MET + EX-527 was higher than in MET alone and approached the TBHP level (Fig 4c). Collectively, these findings indicate that MET mitigates oxidative stress and apoptosis in LECs at least partly via activation of the SIRT1/FOXO1 pathway, as evidenced by EX-527–sensitive effects on ROS, MDA/peroxidase activity, and apoptosis.

### Contribution of SIRT1/FOXO1 signaling to MET-associated autophagy regulation

We then evaluated whether SIRT1/FOXO1 mediates MET’s pro-autophagic effect in LECs by repeating autophagy assays with or without EX-527. Immunofluorescence showed that TBHP suppressed autophagy-related proteins (Beclin-1 and LC3B decreased while p62 increased), whereas MET pretreatment restored Beclin-1 and LC3B and reduced p62; EX-527 co-treatment substantially attenuated these MET-induced changes (Fig 4d-f). Immunoblotting corroborated the imaging results: TBHP decreased Beclin-1 and the LC3B-II/I ratio and increased p62, whereas MET increased Beclin-1, raised LC3B-II/I, and lowered p62 relative to TBHP alone; EX-527 blunted these effects (Fig 4g).

## Discussion

Our data show that metformin pretreatment improves survival and reduces oxidative burden and apoptosis in TBHP-injured HLE-B3 cells, accompanied by EX-527–sensitive changes in SIRT1/FOXO1 signaling and autophagy marker/flux-consistent signatures in vitro. Specifically, MET was associated with higher LC3B conversion and lower p62 under TBHP, and p62 accumulation under transient lysosomal blockade (CQ or BafA1) was compatible with impaired cargo clearance during ongoing autophagosome turnover. Pharmacologic inhibition of early autophagy (3-MA) or SIRT1 (EX-527) partially attenuated MET-associated improvements across oxidative stress and apoptosis endpoints, supporting involvement of autophagy and SIRT1/FOXO1 signaling in this response. Because the mechanistic inference is based on pharmacologic perturbation and marker-based/lysosomal-blockade readouts, the results should be interpreted as supportive and hypothesis-strengthening rather than as definitive proof of a single linear pathway.

These findings align with prior work while refining the focus toward antioxidant/autophagy mechanisms. Chen et al. [8] reported that oxidative stress suppresses autophagy in LECs and that MET restores autophagic flux via AMPK activation to mitigate H_2_O_2_-induced injury; in vivo, low-dose MET preserved lens transparency through AMPK-related mechanisms [9]. While those studies emphasized AMPK signaling and, in some settings, aging-related outcomes, our data highlight SIRT1/FOXO1 as an additional, inhibitor-sensitive axis in human LECs and—at the level of dynamics—demonstrate that MET promotes flux rather than static accumulation of autophagosomal markers. Consistently, Fu et al. [12] reported that under hyperglycemia MET upregulated SIRT1 and restored autophagy in LECs. Together, these studies suggest that AMPK- and SIRT1/FOXO1-linked mechanisms may operate in parallel or sequentially to reinforce autophagy under oxidative pressure.

Mechanistically, MET increased SIRT1, reduced Ac-FOXO1, and normalized autophagy-related proteins, thereby promoting flux. Under lysosomal blockade, the combination of p62 recovery with sustained LC3B conversion is a canonical readout of elevated throughput, integrating molecular changes at the process level. The sensitivity of both LC3B-II/I and p62 to SIRT1 inhibition is consistent with a model in which MET enhances SIRT1 activity, facilitates FOXO1 deacetylation/activation, and supports transcriptional programs that maintain autophagy flux during oxidative stress [13,14].

An additional observation is that MET’s antioxidant profile in this system appears selective. MET lowered ROS, malondialdehyde, and peroxidase activity (MPO-kit readout) and restored SOD activity, yet GSH depletion was not reversed. This discrepancy implies that SIRT1/FOXO1 signaling may preferentially reinforce enzymatic antioxidant defenses (e.g., SOD), whereas glutathione synthesis or recycling is governed by other programs [15]. Such selectivity provides a mechanistic rationale for the incomplete rescue of GSH despite robust effects on ROS handling and apoptosis, and suggests that SIRT1/FOXO1-directed autophagy and enzyme-based detoxification act in concert to re-establish redox homeostasis.

Scope clarification. Our study was designed a priori to delineate antioxidant cytoprotective mechanisms under an acute TBHP paradigm, focusing on ROS burden, apoptosis, and autophagy dynamics (including flux) [16,17]. Classical senescence endpoints (SA-β-Gal, p16/p21, SASP factors) typically require longer recovery windows and a distinct experimental framework; these assays were therefore considered beyond the scope of the present work and are listed in the Limitations as future directions.

We also considered the rationale for our oxidative-stress model. TBHP was chosen over H_2_O_2_ because it is chemically more stable and induces sustained intracellular ROS, providing a reproducible global oxidative injury model. Preliminary assays showed that 500 µM TBHP for 4 h consistently reduced cell viability to ~50%, enabling a robust yet nonlethal stress level. Nevertheless, other inducers (e.g., H_2_O_2_) or mitochondria-targeted stressors (e.g., rotenone or antimycin A) may elicit distinct injury patterns and should be explored in future studies explicitly designed to interrogate those mechanisms [18,19].

Beyond FOXO1, SIRT1 governs additional stress-response pathways. In LECs exposed to UVB, reduced SIRT1 and NFE2L2 (Nrf2) expression impaired antioxidant defenses and accelerated cataract formation, while SIRT1 inhibition further exacerbated the damage [20,21]. These observations position SIRT1 as a central node in lens oxidative defense, acting through FOXO1-mediated autophagy and other antioxidant regulators. Human studies also indicate complex SIRT1 regulation: some report age-related declines [22], whereas others describe elevated aqueous humor or lens tissue levels in cataract patients, possibly as compensatory responses [23,24]. Yet such endogenous changes appear insufficient to halt disease progression. External pharmacologic activation of SIRT1 may therefore be beneficial. Although resveratrol shows protective effects in cataract models [25], MET is inexpensive, widely used, and has an established safety profile, making it an attractive candidate for antioxidant cytoprotection in the lens.

## Conclusions

In summary, metformin pretreatment attenuated TBHP-induced oxidative injury in human lens epithelial cells, lowering ROS and lipid peroxidation indices and reducing apoptosis in vitro. These protective effects were accompanied by EX-527–sensitive SIRT1/FOXO1 signaling changes and autophagy marker/lysosomal-blockade patterns that were consistent with enhanced autophagy dynamics under oxidative stress. Collectively, the findings support a model in which SIRT1/FOXO1-linked autophagy responses may contribute to MET-associated cytoprotection in lens epithelium, warranting further validation in future studies.

### Limitations of this study

This study has several limitations. First, our conclusions are based on an in-vitro oxidative-stress model in a single human lens epithelial cell line (HLE-B3); therefore, the generalizability to primary LECs and in-vivo cataract models remains to be validated. Second, although lysosomal blockade (CQ/BafA1) and multiple autophagy readouts support an association between metformin treatment and improved autophagic flux under TBHP stress, pharmacological inhibitors may have off-target effects, and we did not employ genetic approaches (e.g., SIRT1/FOXO1 manipulation or autophagy-gene perturbation) to establish definitive causality. Third, the study focused on a limited set of time points and one oxidative stimulus, so a broader dose–time response may further refine the therapeutic window. Finally, classical senescence endpoints (SA-β-Gal, p16, p21, SASP factors) were not measured, limiting inference on metformin’s senescence-related actions in LECs.

## Supporting information

S1 Data Raw numerical data underlying all graphs and statistical analyses. (XLSX)

S2 File Original uncropped and unadjusted western blot images corresponding to the blot panels shown in the main figures. (PDF)

## Abbreviations

3-MA: Methyladenine
Ac-FOXO1: Acetylated forkhead box O1
AMPK: AMP-activated protein kinase
ARC: Age-related cataract
ATG: Autophagy-related gene
BafA1: Bafilomycin A1
CQ: Chloroquine
FOXO1: Forkhead box O1
GSH: Glutathione
LC3/LC3B: Microtubule-associated protein 1 light chain 3 (isoform B)
LAMP1: Lysosome-associated membrane protein 1
LECs: Lens epithelial cells
MDA: Malondialdehyde
MDC: Monodansylcadaverine
MET: Metformin
MPO: Myeloperoxidase
Nrf2 (NFE2L2): Nuclear factor erythroid 2–related factor 2
p62: Sequestosome 1
ROS: Reactive oxygen species
SIRT1: Silent information regulator 1
SOD: Superoxide dismutase
TBHP: Tert-butyl hydroperoxide.
