# Supplementary figures and images for: Metformin attenuates TBHP-induced oxidative injury in human lens epithelial cells and is associated with SIRT1/FOXO1-related autophagy
Source: PLoS One. 2026 Apr 7;21(4):e0346822. doi: 10.1371/journal.pone.0346822 (PMC13056204; doi:10.1371/journal.pone.0346822)

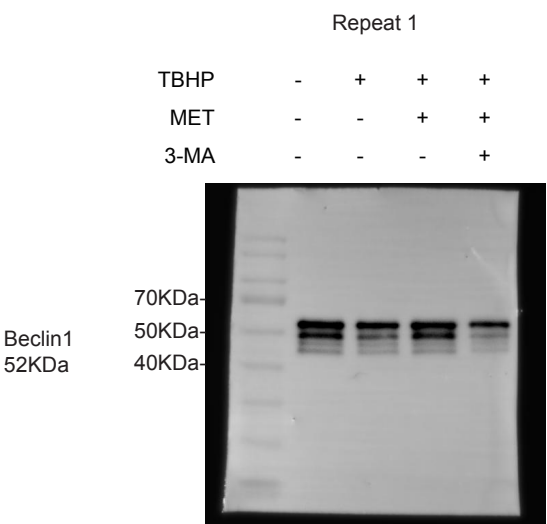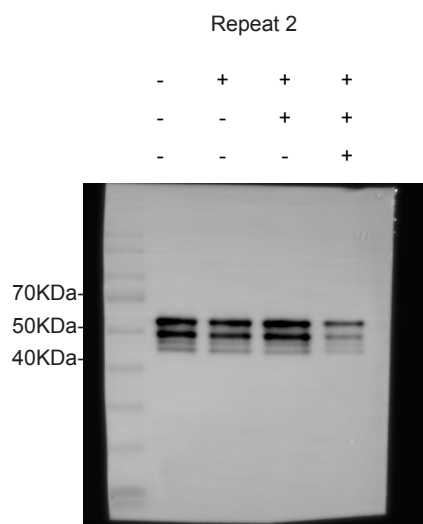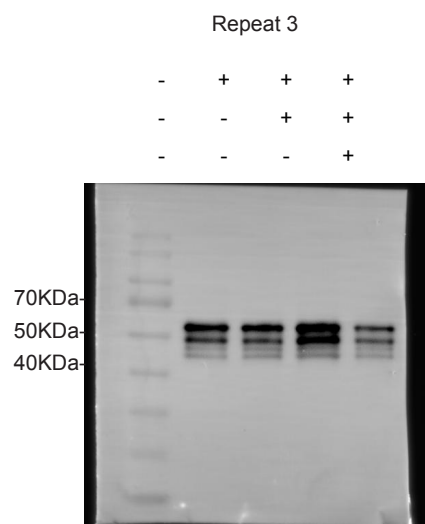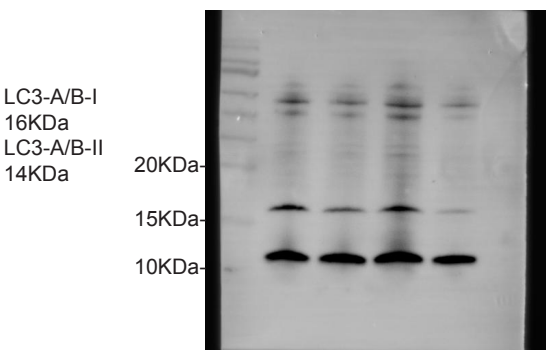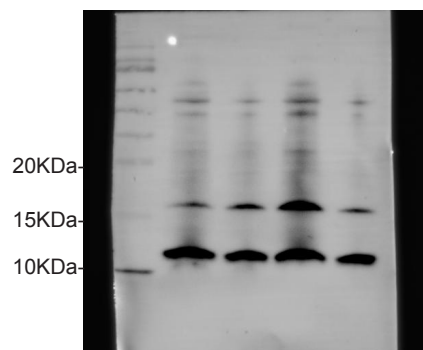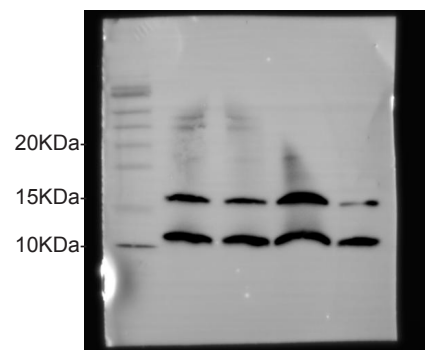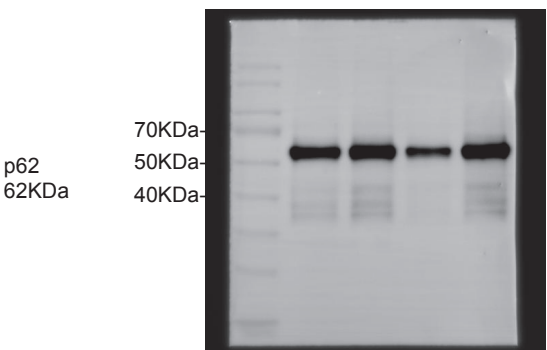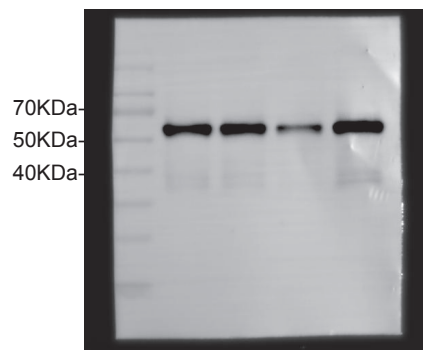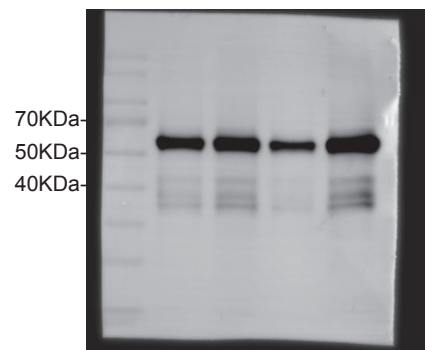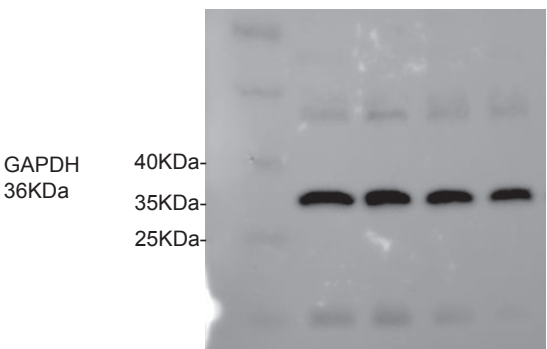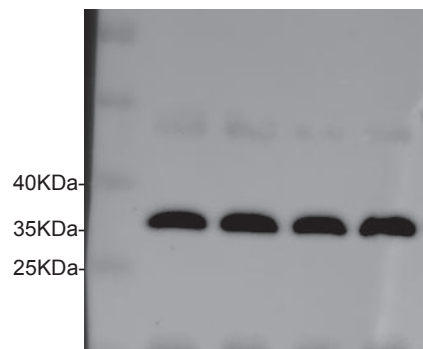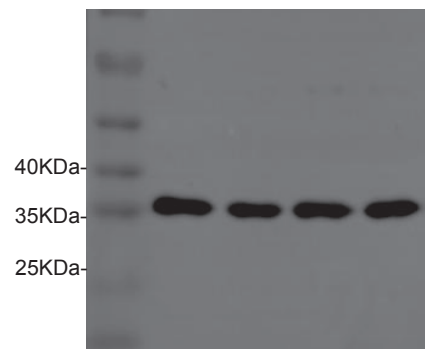

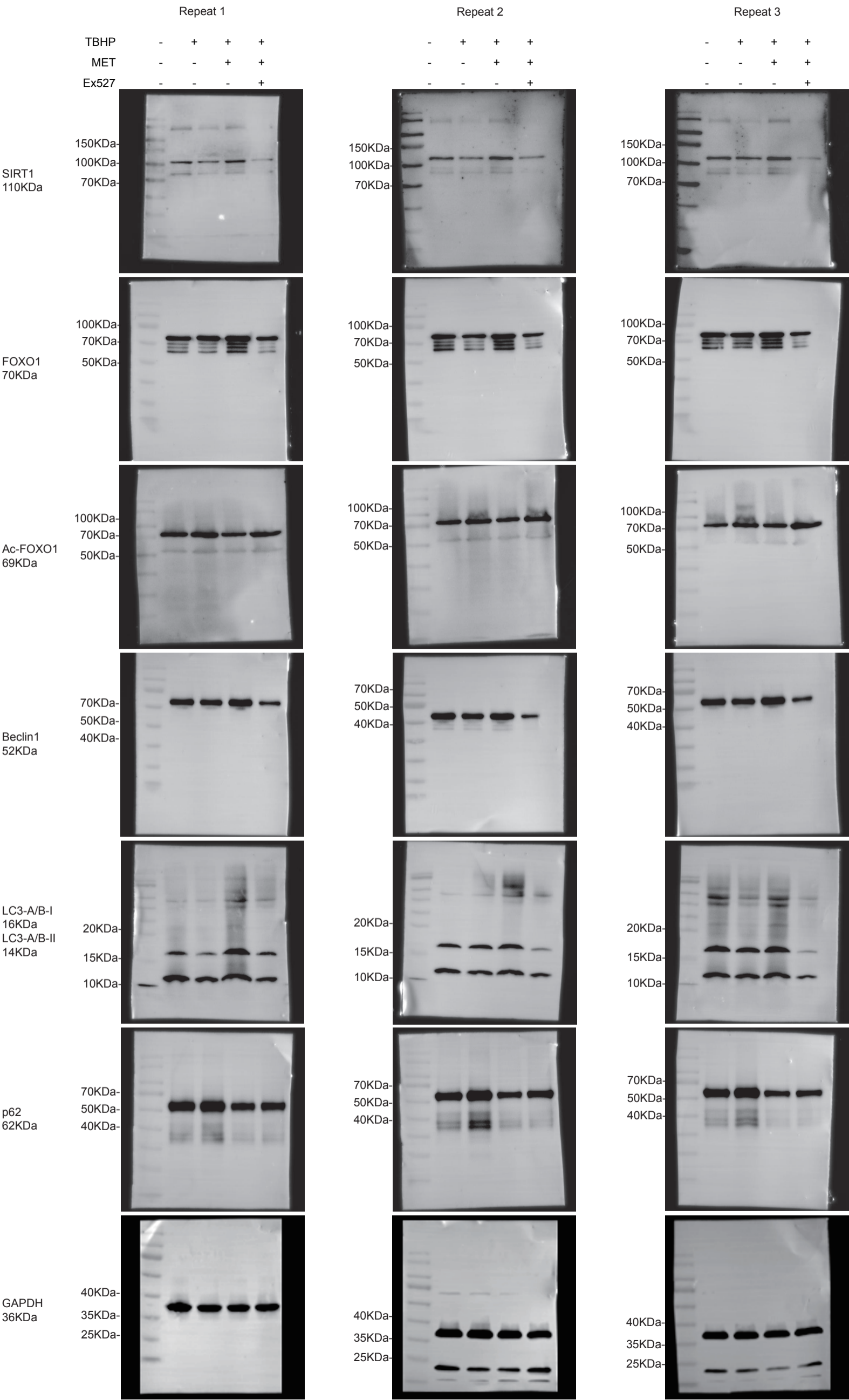

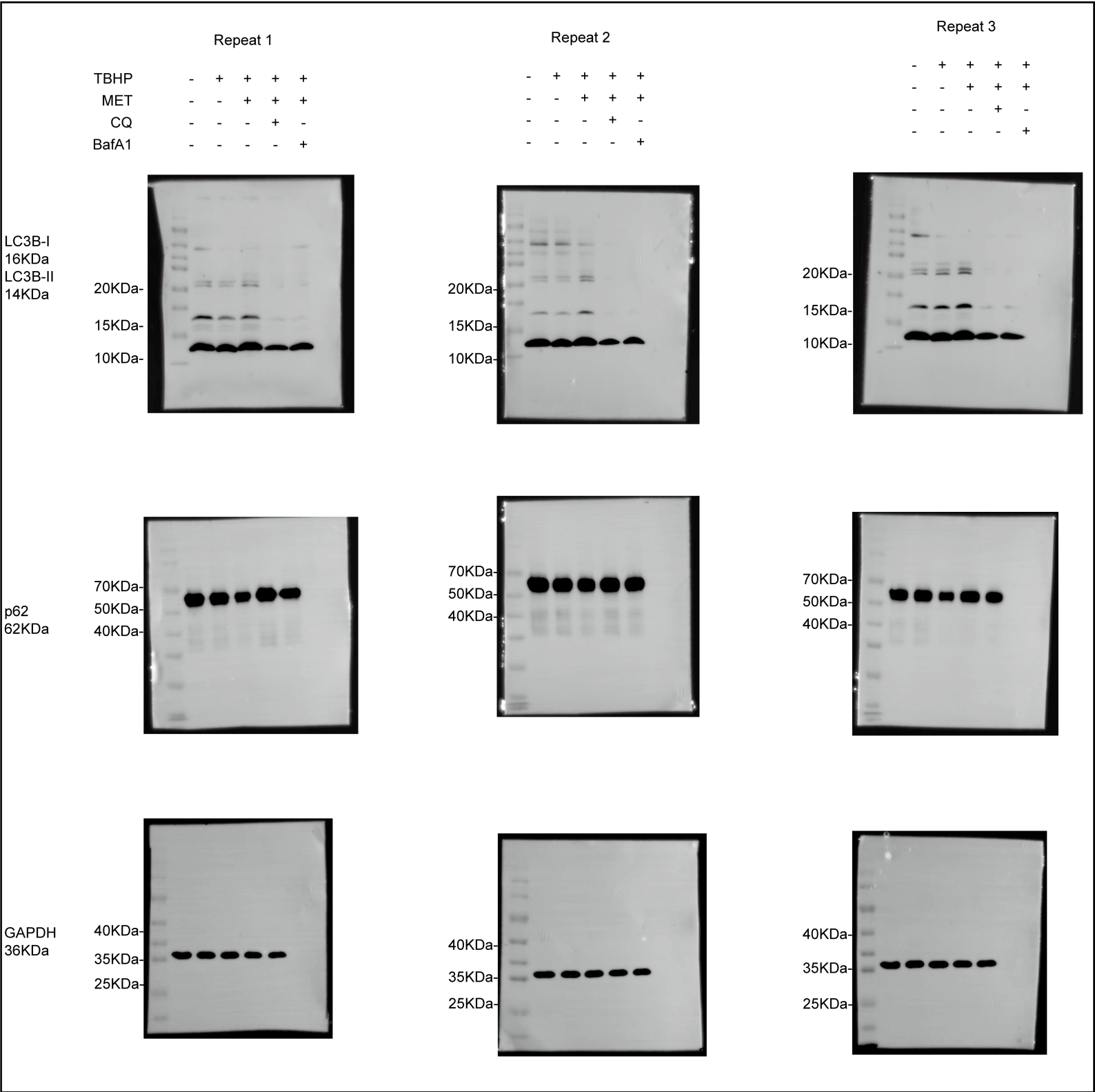

Supplement: S2 File — (PDF) [file pone.0346822.s002.pdf]
